# Translational Repression of the RpoS Antiadapter IraD by CsrA Is Mediated via Translational Coupling to a Short Upstream Open Reading Frame

**DOI:** 10.1128/mBio.01355-17

**Published:** 2017-08-29

**Authors:** Hongmarn Park, Louise C. McGibbon, Anastasia H. Potts, Helen Yakhnin, Tony Romeo, Paul Babitzke

**Affiliations:** a Department of Biochemistry and Molecular Biology, Center for RNA Molecular Biology, The Pennsylvania State University, University Park, Pennsylvania, USA; b Department of Microbiology and Cell Science, Institute of Food and Agricultural Sciences, University of Florida, Gainesville, Florida, USA; Princeton University

**Keywords:** CsrA, IraD, RpoS, translational coupling, translational control

## Abstract

CsrA is a global regulatory RNA binding protein that has important roles in regulating carbon metabolism, motility, biofilm formation, and numerous other cellular processes. IraD functions as an antiadapter protein that inhibits RssB-mediated degradation of RpoS, the general stress response and stationary-phase sigma factor of *Escherichia coli*. Here we identified a novel mechanism in which CsrA represses *iraD* translation via translational coupling. Expression studies with quantitative reverse transcriptase PCR, Western blotting, and *lacZ* fusions demonstrated that CsrA represses *iraD* expression. Gel mobility shift, footprint, and toeprint studies identified four CsrA binding sites in the *iraD* leader transcript, all of which are far upstream of the *iraD* ribosome binding site. Computational modeling and RNA structure mapping identified an RNA structure that sequesters the *iraD* Shine-Dalgarno (SD) sequence. Three open reading frames (ORFs), all of which are translated, were identified in the *iraD* leader region. Two of these ORFs do not affect *iraD* expression. However, the translation initiation region of the third ORF contains three of the CsrA binding sites, one of which overlaps its SD sequence. Furthermore, the ORF stop codon overlaps the *iraD* start codon, a sequence arrangement indicative of translational coupling. *In vivo* expression and *in vitro* translation studies with wild-type and mutant reporter fusions demonstrated that bound CsrA directly represses translation initiation of this ORF. We further established that CsrA-dependent repression of *iraD* translation occurs entirely via translational coupling with this ORF, leading to accelerated *iraD* mRNA decay.

## IMPORTANCE

CsrA posttranscriptionally represses gene expression associated with stationary-phase bacterial growth, often in opposition to the transcriptional effects of the stationary-phase sigma factor RpoS. We show that CsrA employs a novel regulatory mechanism to repress translation of *iraD*, which encodes an antiadapter protein that protects RpoS against proteolysis. CsrA binds to four sites in the *iraD* leader transcript but does not directly occlude ribosome binding to the *iraD* SD sequence. Instead, CsrA represses translation of a short open reading frame encoded upstream of *iraD*, causing repression of *iraD* translation via translational coupling. This finding offers a novel mechanism of gene regulation by the global regulator CsrA, and since RpoS can activate *csrA* transcription, this also highlights a new negative-feedback loop within the complex Csr and RpoS circuitry.

## INTRODUCTION

Bacteria sense and respond to environmental signals via global regulatory networks, which coordinate sweeping changes in gene expression. The Csr system, also called Rsm in some organisms, is one such network that globally controls gene expression posttranscriptionally. Depending on the particular species, the Csr/Rsm system regulates a variety of cellular processes, including carbon metabolism, motility, biofilm formation, quorum sensing, and virulence (1). CsrA, the central component of the *Escherichia coli* Csr system, is a homodimeric RNA binding protein containing two identical RNA binding surfaces. These two surfaces can simultaneously bind to two sites within a target transcript (2, 3). GGA is a critical motif in CsrA binding sites and is often present in the loop of short RNA hairpins (4, 5). CsrB and CsrC (CsrB/C) are two small RNAs (sRNAs) that function by antagonizing CsrA activity; each sRNA contains several CsrA binding sites and is capable of sequestering multiple CsrA dimers (4, 6). Transcription of *csrB* and *csrC* is activated by the BarA-UvrY two-component signal transduction system in response to short-chain-carboxylic-acid-containing metabolites (7, 8) and by ppGpp (9, 10). A membrane-bound GGDEF-EAL domain protein, CsrD, is required for initiation of CsrB/C decay by RNase E cleavage (11, 12). Glucose activates CsrB/C decay by binding of CsrD to the unphosphorylated form of EIIA^Glc^, which predominates during glucose uptake by the phosphotransferase system (PTS) (13). Thus, the availability of preferred carbon favors depressed CsrB/C levels and increased CsrA availability, while the combination of depletion of preferred carbon and accumulation of end products causes CsrB/C accumulation and CsrA sequestration. In addition, transcription of *csrA* is driven by five promoters, two of which utilize RpoS (σ^S^) (14), the general stress response and stationary-phase sigma factor of *E. coli*. CsrA can also repress its own translation by binding to four sites in the leader RNA, one of which overlaps its Shine-Dalgarno (SD) sequence, thereby directly competing with ribosome binding (14). Thus, the level of available CsrA is tightly controlled in the cell. Negative feedback within the Csr circuitry is mediated via indirect CsrA-dependent activation of CsrB/C synthesis (15) and repression of *csrD* (11) and has been found to improve response times and signaling dynamics in the Csr system (16).

A global study employing transcriptome sequencing (RNA-seq) previously identified several hundred mRNAs bound by CsrA (9). In two cases, it was shown that bound CsrA alters the structure surrounding the SD sequence, leading to repression or activation of expression by affecting translation efficiency (17, 18). However, the most common CsrA-mediated regulatory mechanism involves CsrA binding to multiple GGA motif-containing sites, one of which overlaps the cognate SD sequence, such that bound CsrA represses translation initiation by directly occluding the ribosome binding site (1). Although in some instances CsrA binds with high affinity to the site overlapping the SD sequence, in other cases one RNA binding surface of the CsrA dimer binds to a high-affinity site, thereby bridging the other surface to a lower affinity site overlapping the SD sequence (3, 19). In addition, CsrA-mediated translational repression often leads to destabilization of the mRNA because the mRNA is then accessible to RNases (1, 19).

It is well documented that gene expression can be controlled by translational coupling, a process in which translation of a downstream cistron is at least partially dependent on translation of the cistron immediately upstream (20, 21). In some coupling mechanisms, translation of the upstream cistron disrupts an SD-sequestering hairpin that would otherwise block translation of the downstream cistron, while other coupling mechanisms involve overlapping stop and start codons of the two cistrons.

**FIG 1:**
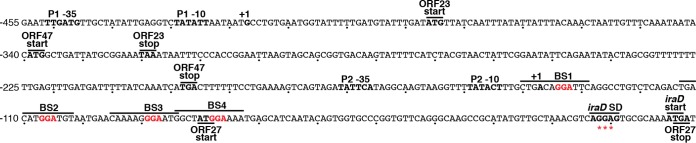
Nucleotide sequence of the *iraD* promoter and leader region. −35 and −10 elements of promoters P1 and P2 and the corresponding transcription start sites (+1) are in bold. The ORF23, ORF47, and ORF27 translation start and stop codons are in bold type and marked with lines. The *iraD* Shine-Dalgarno (SD) sequence and start codon are also marked. Authenticated CsrA binding sites BS1 to BS4 are marked with a line, with critical GGA motifs in red. A fifth GGA motif (indicated by three asterisks [***]) overlaps the *iraD* SD sequence, but we determined in this study that CsrA does not bind to this region of the transcript. Numbering is with respect to the start of *iraD* translation.

IraD functions as an antiadapter protein that prevents RssB adapter-mediated degradation of RpoS by the ClpXP protease (22). Induction of IraD expression occurs in response to DNA damage and during the transition to stationary phase via ppGpp-dependent promoters P1 and P2. Transcription from P1 occurs with RNA polymerase (RNAP) containing σ^70^, whereas transcription from P2 occurs with RNAP containing either σ^70^ or σ^S^ (Fig. 1) (23, 24). In this study, we found that CsrA regulates *iraD* expression by binding to *iraD* leader RNA. The *iraD* leader forms a strong SD-sequestering structure. In addition, an open reading frame (ORF) was identified whose stop codon overlaps the *iraD* start codon. We demonstrate that CsrA represses translation initiation of the ORF, leading to translational repression of *iraD*, exclusively by preventing translational coupling with the ORF. This finding expands the known interactions between two global regulators of stationary-phase gene expression and stress responses, CsrA and RpoS, and reveals a new feedback loop of the Csr circuitry.

## RESULTS

### CsrA represses *iraD* expression.

**FIG 2:**
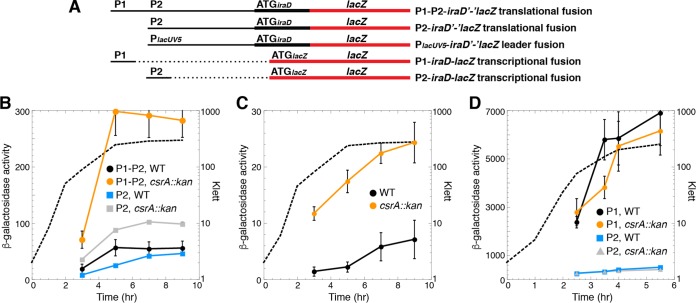
CsrA represses *iraD* expression. (A) Schematic representation of the fusions used in this analysis. Relative positions of the *iraD* promoters (P1 and P2), P_*lacUV5*_, and the start codons (ATG) driving translation of each fusion are shown. The region that includes the *iraD* promoter and leader is depicted with a thin black line, while the *iraD* and *lacZ* coding sequences are depicted with thick black and red lines, respectively. Dashed lines indicate that the corresponding sequence is absent. (B to D) β-Galactosidase activities (in Miller units) ± standard errors were determined throughout growth. Experiments were performed at least three times. Representative growth curves are shown with dashed lines (Klett). (B) Expression of P1-P2-*iraD*'*-*'*lacZ* and P2-*iraD*'*-*'*lacZ* translational fusions in wild-type (WT) and *csrA*::*kan* mutant strains. (C) Expression of the P*_lacUV5_-iraD*'*-*'*lacZ* leader fusion in WT and *csrA*::*kan* mutant strains. (D) Expression of P1-P2-*iraD-lacZ* and P2-*iraD-lacZ* transcriptional fusions in WT and *csrA*::*kan* mutant strains.

**FIG 3:**
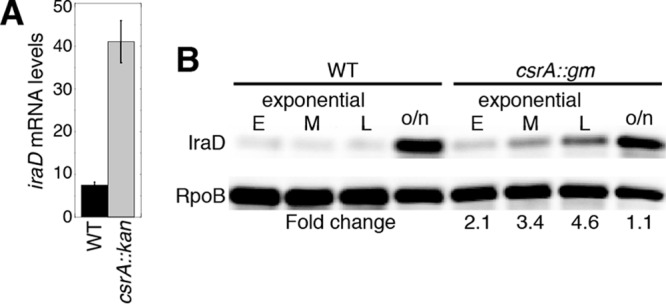
CsrA represses *iraD* mRNA and IraD protein levels. (A) qRT-PCR analysis of *iraD* mRNA levels in WT and *csrA*::*kan* mutant strains at mid-exponential phase. Values represent averages of results from 3 experiments ± standard deviations. (B) Western blot analysis of IraD levels in WT and *csrA*::*gm* strains grown to early (E), mid (M), and late (L) exponential phase. o/n, overnight cultures. Fold change values (*csrA*::*gm*/WT) are shown. Values are normalized to RpoB levels. Results are representative of two independent experiments.

Visual inspection of the *iraD* leader region led to the identification of five potential CsrA binding sites, each containing a critical GGA motif (Fig. 1). We first examined expression of P1-P2-*iraD*'*-*'*lacZ* and P2-*iraD*'*-*'*lacZ* translational fusions in wild-type (WT) and CsrA-deficient (*csrA*::*kan*) strains. This *csrA*::*kan* allele contains a transposon insertion near the C-terminal coding sequence of *csrA*, resulting in a mutant CsrA protein that retains about 12% of the binding activity observed in the WT protein (25). Expression of both fusions in the WT background was low during exponential growth and then increased as cells entered stationary phase (Fig. 2A and B). Notably, in the *csrA*::*kan* mutant background, the levels of expression of the P1-P2-*iraD*'*-*'*lacZ* and P2-*iraD*'*-*'*lacZ* translational fusions were elevated about 7- and 3-fold, respectively, indicating that CsrA represses *iraD* expression. We also examined expression of a leader fusion in which the *iraD* P1-P2 promoter region was replaced with the *lacUV5* promoter (P_*lacUV5*_) (Fig. 2A and C). Since CsrA does not affect transcription from P_*lacUV5*_ (9), an effect of the *csrA*::*kan* mutation is inferred to reflect CsrA-dependent posttranscriptional effects of the *iraD* leader RNA. As was seen with the P2-*iraD*'*-*'*lacZ* translational fusion, expression of the P_*lacUV5*_-*iraD*'*-*'*lacZ* leader fusion was ~3-fold higher in the *csrA*::*kan* strain (Fig. 2C). When similar experiments were performed with P1-*iraD*-*lacZ* and P2-*iraD-lacZ* transcriptional fusions in which the *iraD* leader region was absent, expression levels were similar in WT and *csrA*::*kan* strains, confirming that CsrA-dependent regulation of *iraD* expression is independent of the *iraD* promoters (Fig. 2A and D). We also performed quantitative reverse transcriptase PCR (qRT-PCR) experiments and found that *iraD* mRNA levels were 5-fold higher in the *csrA*::*kan* strain (Fig. 3A). In addition, a Western blot demonstrated that IraD protein levels increased ~5-fold in the *csrA*::*kan* strain during exponential phase (Fig. 3B). However, IraD reached the same levels in WT and *csrA*::*kan* strains in overnight cultures, suggesting that the physiological role of CsrA is to prevent premature accumulation of IraD. Taken together, these data suggest that CsrA directly represses *iraD* expression posttranscriptionally.

### CsrA binds specifically to *iraD* leader RNA.

**FIG 4:**
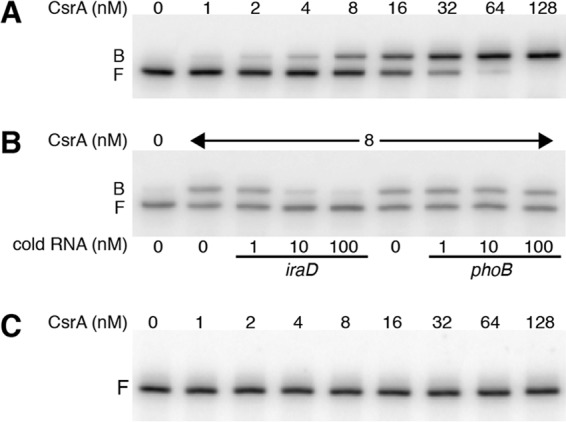
Gel mobility shift analysis of CsrA-*iraD* leader RNA interaction. (A to C) Labeled *iraD* RNA (0.1 nM) was incubated with the concentration of CsrA shown at the top of each lane. Positions of bound (B) and free (F) RNA are marked. Experiments were performed at least twice, and representative gels are shown. (A) CsrA forms a single high-affinity complex with WT *iraD* RNA. (B) RNA competition experiment demonstrating binding specificity. Labeled *iraD* RNA was incubated with the indicated concentration of unlabeled specific (*iraD*) or nonspecific (*phoB*) competitor RNA. (C) CsrA binding to *iraD* RNA is eliminated when the GGA motifs in BS2, BS3, and BS4 are changed to CCA.

Quantitative gel mobility shift assays were performed to determine whether CsrA bound to an *iraD* transcript extending from the P2 transcription start site into the early *iraD* coding sequence. Although this transcript contained five potential CsrA binding sites (Fig. 1), only one shifted complex was observed. A distinct band was observed between 2 and 128 nM CsrA, indicating that CsrA formed a tight complex with this transcript with an apparent *K*_*d*_ (dissociation constant) value of 15 nM (Fig. 4A), which is similar to the values seen with other known mRNA targets (1, 3, 14, 19). The specificity of CsrA-*iraD* RNA interaction was investigated by performing competition experiments with specific (*iraD*) and nonspecific (*phoB*) unlabeled RNA competitors. Unlabeled *iraD* was an effective competitor, whereas *phoB* was not (Fig. 4B). We next tested binding to a transcript in which the GGA motifs of BS2, BS3, and BS4 were all changed to CCA. These mutations eliminated CsrA binding (Fig. 4C). We conclude that CsrA binds to *iraD* with high affinity and specificity and that BS2, BS3, and/or BS4 function as CsrA binding sites.

**FIG 5:**
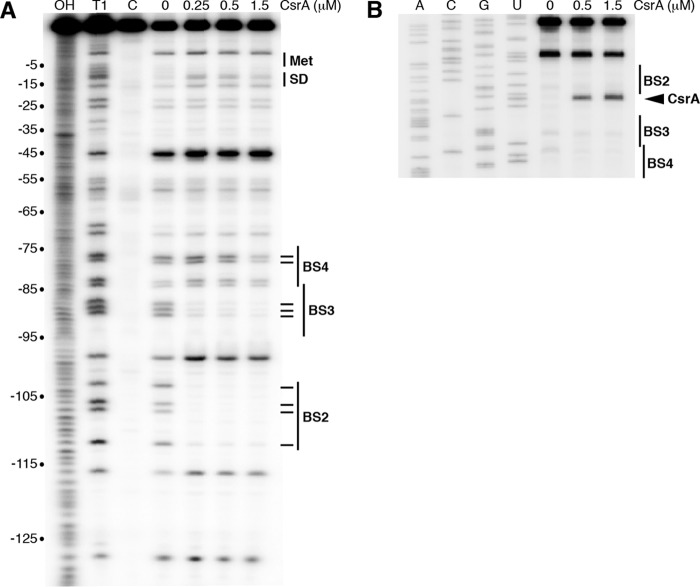
CsrA-*iraD* RNA footprint and toeprint analyses. (A) CsrA-*iraD* RNA footprint. Labeled *iraD* RNA was treated with RNase T1 in the presence of the CsrA concentration shown at the top of the lane. Partial alkaline hydrolysis (OH) and RNase T1 digestion (T1) ladders, as well as a control lane without RNase T1 treatment (C), are marked. Positions of BS2, BS3, BS4, the *iraD* start codon (Met), and the Shine-Dalgarno (SD) sequence are shown. Residues that were protected by bound CsrA from RNase T1 cleavage are marked (–). Numbering is with respect to the start of *iraD* translation. (B) CsrA-*iraD* RNA toeprint. The concentration of CsrA used is shown at the top of the lane. Positions of BS2, BS3, BS4, and the CsrA toeprint (carat) are marked. Lanes corresponding to results of sequencing to reveal A, C, G, and U residues are labeled.

CsrA-*iraD* RNA footprint experiments using RNase T1 as a single-strand G-specific probe were performed to identify the authentic CsrA binding sites in the *iraD* leader transcript. Bound CsrA resulted in strong protection of RNase T1-mediated cleavage of the G residues between −112 and −103 (BS2) and between −90 to −84 (BS3) and in weak protection at −78 and −77 (BS4) (Fig. 5A). Notably, bound CsrA did not protect the GGA sequence within the *iraD* SD sequence. We also performed CsrA toeprint experiments as an alternative method to observe positions of bound CsrA. A strong CsrA-dependent toeprint band was observed just downstream from BS2, whereas toeprints were not observed downstream from the other binding sites, suggesting that CsrA has the highest affinity for BS2 (Fig. 5B).

Since the transcript in this analysis began at the P2 transcription start site, we were unable to obtain information for BS1 because the GGA motif was too close to the 5′ end of the transcript. Thus, footprint experiments were performed with a transcript whose 5′ end was extended 17 nucleotides (nt) upstream of the P2 transcription start site, which would mimic a transcript originating from P1. In this case, we observed weak protection of BS1 and BS4, strong protection of BS2 and BS3, and no protection of the GGA motif overlapping the *iraD* SD sequence (see Fig. S1 in the supplemental material). We also performed a footprint experiment with RNA containing a GGA-to-CGA mutation in BS2. In addition to complete loss of protection of BS2, this mutation caused a small reduction in the level of protection of BS3 (Fig. S1). We conclude that CsrA is capable of binding to four sites in the *iraD* leader RNA, with tight binding to BS2 and BS3.

10.1128/mBio.01355-17.1 FIG S1  CsrA-*iraD* RNA footprint analysis. Wild-type (WT) or BS2 mutant *iraD* RNA was treated with RNase T1 in the presence of the CsrA concentration shown at the top of the lane. Partial alkaline hydrolysis (OH) and RNase T1 digestion (T1) ladders, as well as a control lane without RNase T1 treatment (c), are shown. Positions of BS1, BS2, BS3, BS4, the *iraD* start codon (Met), and the Shine-Dalgarno (SD) sequence are shown. Residues that are protected by bound CsrA from RNase T1 cleavage are marked (–). Numbering is with respect to the start of *iraD* translation. The position of the BS2 GGA-to-CGA mutation is marked with a carat. Positions in which CsrA-mediated protection was lost in the mutant transcript are marked (*). Sequencing lanes revealing A, C, G, and U residues are labeled. Download FIG S1, TIF file, 3.4 MB. Copyright © 2017 Park et al. 2017 Park et al. This content is distributed under the terms of the Creative Commons Attribution 4.0 International license.

### BS2 is critical for CsrA-mediated repression of *iraD*.

**FIG 6:**
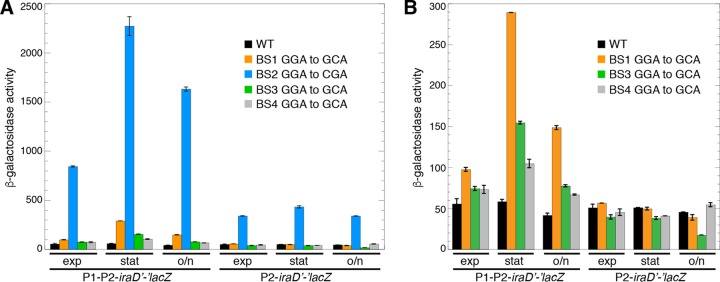
Effects of CsrA binding site mutations (BS1, BS2, BS3, and BS4) on expression of P1-P2-*iraD*'*-*'*lacZ* and P2-*iraD*'*-*'*lacZ* translational fusions. (A and B) β-Galactosidase activities (Miller units) ± standard deviations were determined in exponential-phase (exp), stationary-phase (stat), and overnight (o/n) cultures. Experiments were performed at least three times. (A) Wild-type (WT) and mutant fusions containing the indicated changes in BS1, BS2, BS3, and BS4. (B) WT and mutant fusions containing the indicated changes in BS1, BS3, and BS4 to emphasize small differences caused by these mutations.

Since our gel shift assay indicated that mutating BS2, BS3, and BS4 eliminated CsrA binding, we tested the effect of single nucleotide substitutions in BS1 (GGA to GCA), BS2 (GGA to CGA), BS3 (GGA to GCA), or BS4 (GGA to GCA) in our reporter assay. The BS1 mutation resulted in elevated expression of the P1-P2-*iraD*'*-*'*lacZ* translational fusion but not the P2-*iraD*'*-*'*lacZ* translational fusion (Fig. 6), indicating that BS1 functions as a CsrA binding site only in transcripts that originate from P1, presumably because the GGA motif is too close to the 5′ end of transcripts originating from P2; there are only 3 nt upstream of this GGA motif in transcripts that initiate from P2 (Fig. 1). This interpretation is consistent with the systematic evolution of ligands by exponential enrichment (SELEX)-derived CsrA binding site consensus sequence containing 6 nt upstream and 3 nt downstream from the GGA motif (5). The mutation in BS2 resulted in a dramatic increase in expression of both the P1-P2-*iraD*'*-*'*lacZ* and P2-*iraD*'*-*'*lacZ* translational fusions throughout all stages of growth (Fig. 6A). In contrast, the BS3 and BS4 mutations resulted in only a modest increase in expression of the P1-P2-*iraD*'*-*'*lacZ* fusion, particularly in stationary phase (Fig. 6B). We also noted a modest decrease in expression of the P2-*iraD*'*-*'*lacZ* translational fusion containing the BS3 mutation in the overnight culture (Fig. 3B). These results for BS3 were surprising in the context of the footprint data showing that CsrA bound to both BS2 and BS3 with high affinity (Fig. 5A). This discrepancy is addressed below.

**FIG 7:**
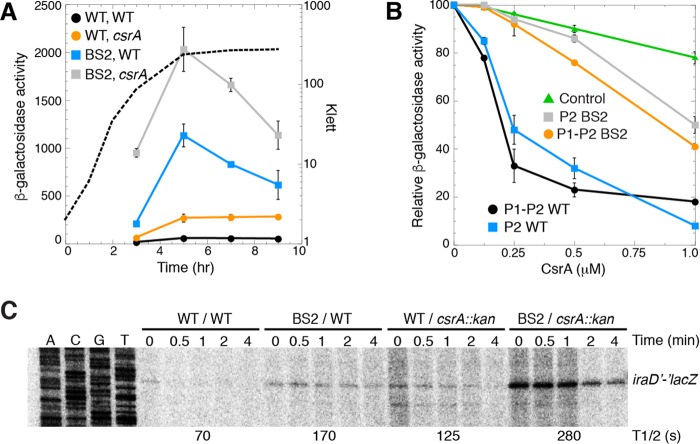
Effects of *csrA*::*kan* and BS2 mutations on *iraD* expression. (A) β-Galactosidase activities (Miller units) ± standard deviations of P1-P2-*iraD*'*-*'*lacZ* translational fusions were determined throughout growth. Experiments were performed at least three times. A representative growth curve is shown with a dashed line (Klett). Symbols: black, WT fusion WT *csrA* strain; orange, WT fusion *csrA*::*kan* strain; blue, BS2 mutant fusion WT *csrA* strain; gray, BS2 mutant fusion *csrA*::*kan* strain. (B) β-Galactosidase activities ± standard deviations of P1-P2-*iraD*'*-*'*lacZ* and P2-*iraD*'*-*'*lacZ* translational fusions in the presence of the indicated CsrA concentration were determined usinjg a PURExpress system. Experiments were performed at least three times. Values for samples without CsrA were set to 100. Symbols: black, WT P1-P2-*iraD*'*-*'*lacZ* fusion; orange, BS2 mutant P1-P2-*iraD*'*-*'*lacZ* fusion; blue, WT P2-*iraD*'*-*'*lacZ* fusion; gray, BS2 mutant P2-*iraD*'*-*'*lacZ* fusion; green, control *pnp*'*-*'*lacZ* translational fusion that is not repressed by CsrA (19). (C) Cultures were grown to mid-exponential phase prior to the addition of rifampin. Samples were harvested at the indicated times and then analyzed by primer extension for *iraD*'*-*'*lacZ* mRNA levels. mRNA half-lives (T1/2) are shown at the bottom of the gel. This experiment was performed twice.

Since BS2 was critical for repression of *iraD* expression, we examined the effect of this binding site in more detail. First, we compared the levels of expression of the WT and BS2 mutant P1-P2-*iraD*'*-*'*lacZ* translational fusions in WT and *csrA*::*kan* strains. While the BS2 mutation resulted in a more dramatic increase in expression than the *csrA*::*kan* mutation, the BS2 *csrA*::*kan* mutant combination exhibited the highest level of expression (Fig. 7A). These results indicate that despite complete loss of binding to BS2 as determined by footprinting, CsrA is still capable of repressing expression of the fusion by binding to one or more of the remaining sites. Furthermore, the finding that *iraD* expression was not fully derepressed by the *csrA*::*kan* mutation is consistent with the mutant CsrA protein encoded by the *csrA*::*kan* allele retaining ~12% of its WT RNA binding activity (25).

We next used an *in vitro* coupled transcription-translation PURExpress system to determine whether CsrA represses *iraD* translation. Four different plasmids carrying *iraD*'*-*'*lacZ* translational fusions were used in this analysis, all of which were driven by identical T7 RNAP promoters. Two plasmids gave rise to transcripts that initiated at the P1 transcription start site, while the other two plasmids gave rise to transcripts that initiated at the P2 transcription start site. In each case, one of the plasmids contained the WT sequence and the other contained the BS2 mutation. Addition of CsrA in increasing concentrations resulted in 80% to 90% repression of the WT fusions, whereas only 50% repression was observed for the BS2 mutant fusions (Fig. 7B). A *pnp*'*-*'*lacZ* translational fusion that we had previously shown was not repressed by CsrA (19) served as a negative control. These results indicate that CsrA represses translation of *iraD* despite the fact that the 3′ end of the closest binding site (BS4) is 58 nt upstream from the *iraD* SD sequence (Fig. 1).

Since translational repression often leads to destabilization of mRNA, we determined the *iraD* mRNA half-life of the WT and BS2 mutant P1-P2-*iraD*'*-*'*lacZ* translational fusions in WT and *csrA*::*kan* strains. Since we were unable to detect the transcript of the WT fusion in the WT (*csrA*^+^) genetic background by Northern analysis, we performed primer extension experiments using a primer that hybridized to *lacZ* just downstream of the fusion junction. We found that the *csrA*::*kan* and BS2 mutations increased the *iraD* mRNA half-life (Fig. 7C), suggesting that CsrA-mediated translational repression reduces *iraD* mRNA stability.

### Two ORFs that do not affect *iraD* expression are expressed in the *iraD* leader.

We identified sequences capable of encoding ORFs of 23 and 47 amino acids (aa) in the 280-nt stretch that lies between the P1 and P2 transcription start sites (Fig. 1, ORF23 and ORF47). To test whether either of these ORFs was expressed, we generated P1-ORF23'*-*'*lacZ* and P1-ORF47'*-*'*lacZ* translational fusions. Both of these fusions were expressed, with ORF23 expression being higher (Fig. S2A). To test whether expression of ORF23 or ORF47 affected expression of *iraD*, we changed each of the start codons to stop codons independently in the context of the P1-P2-*iraD*'*-*'*lacZ* translational fusion. Since expression of the mutant fusions was comparable to that seen with the WT fusion, we conclude that expression of these ORFs did not affect *iraD* expression (Fig. S2B). Thus, we did not explore these ORFs further.

10.1128/mBio.01355-17.2 FIG S2  Expression of ORF23 and ORF47 does not affect *iraD* expression. (A and B) β-Galactosidase activity data (Miller units) ± standard deviations were determined throughout growth. Experiments were performed at least three times. Representative growth curves are shown with dashed lines (Klett). (A) Expression of P1-ORF23′*-*′*lacZ* and P1-ORF47′*-*′*lacZ* translational fusions. (B) Expression of wild-type (WT) or mutant P1-P2-*iraD*′*-*′*lacZ* translational fusions. Stop indicates that the fusion contains a stop codon soon after translation initiation of ORF23 or ORF47. Download FIG S2, TIF file, 3.6 MB. Copyright © 2017 Park et al. 2017 Park et al. This content is distributed under the terms of the Creative Commons Attribution 4.0 International license.

### CsrA-mediated translational repression of a third *iraD* leader ORF regulates *iraD* expression via translational coupling.

**FIG 8:**
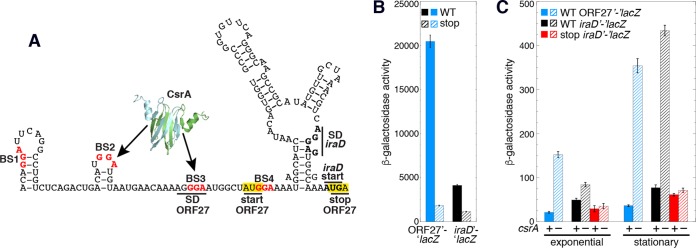
CsrA represses translation of ORF27 and *iraD* via translation coupling. (A) Translational coupling model. CsrA-mediated repression of ORF27 translation leads to repression of *iraD* translation via translational coupling. Critical GGA motifs of CsrA binding sites BS1 to BS4 (red) and the *iraD* start codon are also marked. The ORF27 start and stop codons are boxed in yellow. The ORF27 and *iraD* SD sequences are marked. (B) β-Galactosidase activities ± standard deviations of WT and stop codon mutant ORF27'*-*'*lacZ* and *iraD*'*-*'*lacZ* translational fusions determined *in vitro* with PURExpress. "stop" indicates an ORF27 start-to-stop codon mutation. Experiments were performed at least three times. Values are reported as (1,000)(optical density at 420 nm [OD_420_]) in a 12.5-min reaction. Symbols: solid blue, wild-type (WT) ORF27'*-*'*lacZ* fusion; striped blue, stop codon mutant ORF27'*-*'*lacZ* fusion; solid black, WT *iraD*'*-*'*lacZ* fusion; striped black, stop codon mutant *iraD*'*-*'*lacZ* fusion. (C) β-Galactosidase activities (Miller units) ± standard deviations of P1-P2-ORF27'*-*'*lacZ* and P1-P2-*iraD*'*-*'*lacZ* translational fusions determined in WT (+) and *csrA*::*kan* mutant (–) strains during exponential-phase and stationary-phase growth. Experiments were performed at least three times. Symbols: blue, WT ORF27'*-*'*lacZ* fusion; black, WT *iraD*'*-*'*lacZ* fusion; red, *iraD*'*-*'*lacZ* fusion with an ORF27 stop codon mutation.

CsrA has been shown to repress translation of several genes (1), although in all cases at least one CsrA binding site is located in a position in which bound CsrA could directly block 30S ribosome binding or promote formation of an SD-sequestering hairpin. However, in the case of *iraD*, the 3′ end of the closest binding site is 58 nt upstream of the *iraD* SD sequence, a position that is too remote for CsrA to directly occlude ribosome binding. Using a combination of RNA structure predictions with Mfold data (26) and RNA structure mapping information (Fig. 5A, 0 μM CsrA), we identified an RNA secondary structure that sequesters the *iraD* ribosome binding site, including its SD sequence (Fig. 8A). We also identified a 27-aa ORF with an appropriately spaced SD sequence. Importantly, the ORF27 stop codon overlaps the *iraD* start codon, a sequence arrangement typical of translational coupling (Fig. 8A). Moreover, since BS3 overlaps the ORF27 SD sequence, translational coupling would provide an explanation for the unexpected result in which the GGA-to-GCA mutation in BS3 had little to no effect on *iraD* expression despite BS3 being a high-affinity site (Fig. 5A; Fig. 6; Fig. S1). Although we had predicted that this mutation would cause a substantial increase in expression of the *iraD*'*-*'*lacZ* translational fusions due to reduced CsrA binding, this was not observed. However, this mutation also disrupted the ORF27 SD sequence, which in turn would reduce ORF27 translation and therefore translational coupling with *iraD*, ultimately leading to reduced *iraD* expression.

We performed experiments to test a model in which CsrA directly represses translation of ORF27, leading to repression of *iraD* translation via translational coupling. We first employed an *in vitro* PURExpress system to determine whether ORF27 was expressed. A plasmid containing an ORF27'*-*'*lacZ* translational fusion in which a T7 RNAP promoter gave rise to transcripts initiating from the P1 transcription start site was used for this analysis. High-level expression was observed from this fusion, whereas expression was greatly reduced when the ORF27 start codon was replaced with a UAG stop codon (Fig. 8B). The effect of the ORF27 stop codon mutation was also examined in the context of an *iraD*'*-*'*lacZ* translational fusion in which T7 RNAP initiated transcription from the P1 transcription start site. As with the ORF27'*-*'*lacZ* translational fusion, expression of the *iraD*'-'*lacZ* translational fusion was reduced by the ORF27 stop codon mutation (Fig. 8B). From these data, we conclude that ORF27 is expressed and that loss of ORF27 expression reduces expression of *iraD*.

We next determined the effect of CsrA on expression of a P1-P2-ORF27'*-*'*lacZ* translational fusion *in vivo* and found that expression was much higher in the *csrA*::*kan* strain, indicating that bound CsrA represses ORF27 translation (Fig. 8C). We also examined the effect of CsrA and the ORF27 stop codon mutation on expression of the P1-P2-*iraD*'*-*'*lacZ* translational fusion. As was observed previously (Fig. 2B), expression of the WT fusion was higher in the *csrA*::*kan* mutant background, especially in stationary phase (Fig. 8C). We also found that, relative to expression of the WT fusion, introduction of the ORF27 stop codon reduced expression of the P1-P2-*iraD*'*-*'*lacZ* translational fusion, particularly in the *csrA*::*kan* mutant background during stationary phase. The level of expression that remained in the ORF27 stop codon fusion reflects the level of *iraD* translation that occurs in the absence of translational coupling. Taken together, these data indicate that CsrA represses translation initiation of ORF27, leading to repression of *iraD* translation due to the loss of translational coupling. Moreover, the finding that loss of coupling by the ORF27 stop codon mutant had only a minor effect on expression in the WT (*csrA*^+^) background indicates that CsrA tightly represses ORF27 synthesis.

## DISCUSSION

It is well established that RNA binding proteins and RNA structural features present in the 5′ leader region of mRNA can regulate translation initiation (20, 21). In some cases, the bound protein directly competes with ribosome binding (1, 27), while in others the protein promotes formation of an RNA structure that sequesters the SD sequence (17, 27). Since translating ribosomes can protect the mRNA from ribonucleases, translational repression often leads to decreased mRNA stability. In *E. coli*, CsrA represses translation initiation of many genes, typically by binding two or more sites in target transcripts, where one site overlaps the cognate SD sequence and/or initially translated region. In these instances, bound CsrA represses translation by directly occluding 30S ribosome binding (1, 3, 14, 19). In this study, we found that CsrA represses translation of *iraD*, leading to decreased stability of the transcript and reduced IraD protein levels (Fig. 2, 3, and 7). Although CsrA is capable of binding to four sites in the *iraD* leader RNA, tight binding to BS2 is critical for regulation (Fig. 4 to 6; see also Fig. S1 in the supplemental material). Our data also indicate that CsrA binding to BS1 contributes to regulation of transcripts derived from P1 (Fig. 6). Even though CsrA binds tightly to BS3 (Fig. 5), a mutation in BS3 had little effect on *iraD* expression (Fig. 6). The finding that CsrA represses translation of ORF27 provides an explanation for this discrepancy; the BS3 mutation simultaneously disrupts CsrA binding and inhibits ribosome binding by disrupting the ORF27 SD sequence (Fig. 1 and 8). Thus, the opposing consequences of this mutation essentially cancel each other out.

Translational coupling within a polycistronic transcript is a process in which translation of a downstream cistron depends at least partially on translation of a cistron immediately upstream. In some coupling mechanisms, translation of the upstream gene disrupts an SD-sequestering hairpin for the downstream cistron, thereby freeing the SD sequence for translation initiation by another ribosome. In other examples, coupling involves overlapping stop and start codons of the two cistrons, such that following termination of the upstream cistron, the same ribosome can reinitiate translation of the downstream cistron (20). Of particular interest, translation of *iraD* may be controlled by both of these coupling mechanisms (Fig. 8). Translation of ORF27 would disrupt the *iraD* SD-sequestering hairpin such that a different ribosome could initiate translation of *iraD*. Moreover, since the ORF27 stop codon overlaps the *iraD* start codon, the same ribosome could initiate translation of *iraD* once ORF27 synthesis is complete. In this context, CsrA-dependent repression of *iraD* translation is mediated entirely through its ability to directly repress ORF27 translation, which constitutes a novel CsrA-mediated regulatory mechanism.

**FIG 9:**
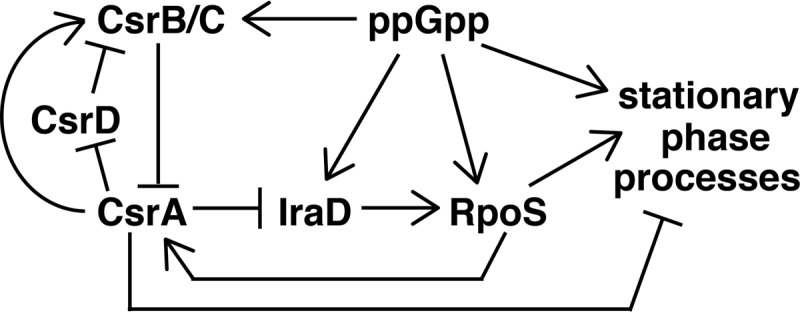
Regulatory circuitry of the Csr, stringent response, and general stress response systems. ppGpp activates transcription of *csrB*, *csrC*, *iraD*, and *rpoS*. CsrA represses IraD synthesis via coupling with ORF27. IraD stabilizes RpoS by inhibiting RssB-mediated degradation of RpoS. RpoS activates transcription of genes involved in stationary-phase processes. RpoS activates transcription of *csrA*, while CsrA represses stationary-phase processes. CsrB/C sRNAs bind to and sequester CsrA from its mRNA targets, while CsrA indirectly activates *csrB*/*C* expression. CsrD targets CsrB/C for degradation by RNase E, and CsrA indirectly represses *csrD* expression. See text for additional details.

RpoS, the general stress response and stationary-phase sigma factor, is controlled by a variety of mechanisms, including regulated proteolysis. IraD functions as an antiadapter protein that binds to the adapter protein RssB, thereby preventing RssB from targeting RpoS for degradation by the ClpXP protease (22). Thus, CsrA-dependent repression of *iraD* translation would lead to increased proteolysis of RpoS (Fig. 9). Furthermore, ppGpp induces *iraD* expression, causing RpoS levels to increase during the stringent response and at the transition to stationary phase (24). Because ppGpp also activates transcription of CsrB/C, the sRNA antagonists of CsrA (9, 10), our finding that CsrA represses *iraD* translation offers a new pathway for increasing IraD and RpoS levels in response to ppGpp, by decreasing CsrA availability. In addition to the connection of CsrA to the general stress response via IraD, RpoS is responsible for activating transcription of *csrA*, leading to increased *csrA* expression during the transition from exponential to stationary phase (14). Thus, *iraD* repression by CsrA apparently creates a negative-feedback loop affecting CsrA and RpoS expression. CsrA activity is also subject to negative feedback through its own effects on *csrB*/*C* transcription and CsrB/C RNA stability, leading to improved signaling response times in the Csr system (11, 15, 16). CsrA, RpoS, and ppGpp play complex roles in coordinating the expression of genes responsible for bacterial lifestyle decisions, e.g., between exponential growth and stationary-phase growth (28–30). The finely tuned interactions between these factors are evidence of a highly evolved regulatory network that is only beginning to be understood.

## MATERIALS AND METHODS

### Bacterial strains and plasmids.

All bacterial strains used in this study are listed in Table 1. *E. coli* strain S17-1 λ *pir+* (31) was used for plasmid construction performed by the conditional-replication, integration, and modular (CRIM) method (32). All numbering throughout the manuscript is with respect to the *iraD* translation initiation codon (Fig. 1). Plasmids pLFT, pLFX, and pUV5 (9) were used to generate translational, transcriptional, and leader fusions, respectively. Plasmid pLM12 contains a P1-P2-*iraD*'*-*'*lacZ* translation fusion (nt −498 to +20 relative to the *iraD* start codon cloned into the PstI and BamHI sites of pLFT). Plasmid pLM13 contains a P2-*iraD*'*-*'*lacZ* translation fusion (nt −234 to +20 cloned into the PstI and BamHI sites of pLFT). Plasmid pLM16 contains a P1-*iraD-lacZ* transcriptional fusion (nt −547 to −413 cloned into the PstI and EcoRI sites of pLFX). Plasmid pLM17 contains a P2-*iraD-lacZ* transcriptional fusion (nt −254 to −133 cloned into the PstI and EcoRI sites of pLFX). Plasmid pLM15 contains a P*_lacUV5_-iraD*'*-*'*lacZ* leader fusion (nt −138 to +20 cloned into the EcoRI and BamHI sites of pUV5) such that the *iraD* P1 and P2 promoter region was replaced with the *lacUV5* promoter. Plasmid pLM30 contains a P1-ORF23'*-*'*lacZ* translational fusion (nt −498 to −376 cloned into the PstI and BamHI sites of pLFT). Plasmid pLM31 contains a P1-ORF47'*-*'*lacZ* translational fusion (nt −498 to −323 cloned into the PstI and BamHI sites of pLFT). Plasmid pHP25 contains a P1-P2-ORF27'*-*'*lacZ* translational fusion (nt −498 to −58 cloned into the PstI and BamHI sites of pLFT). Mutations in the CsrA binding sites (BS1, BS2, BS3, and BS4) in the P1-P2-*iraD*'*-*'*lacZ* and P2-*iraD*'*-*'*lacZ* translational fusions, or mutations of start codons to stop codons in P1-ORF23'*-*'*lacZ*, P1-ORF47'*-*'*lacZ*, and P1-P2-ORF27'-'*lacZ* translational fusions, were introduced using the QuikChange protocol (Agilent Technologies). WT and mutant fusions were integrated into the chromosomal λ att site of strain CF7789 as previously described (32). P1-mediated transduction was used to introduce the *csrA*::*kan* allele from TRMG1655 (7) into strains containing integrated WT and mutant fusions.

**TABLE 1:** *E. coli* strains used in this study

Strain	Description^a^	Source or reference
CF7789	Δ*lacI-lacZ* (MluI)	M. Cashel
MG1655	Prototrophic	M. Cashel
PLB2296	CF7789 P1-P2-*iraD*'*-*'*lacZ* Ap^r^	This study
PLB2297	CF7789 *csrA*::*kan* P1-P2-*iraD*'*-*'*lacZ* Ap^r^ Km^r^	This study
PLB2298	CF7789 P2-*iraD*'*-*'*lacZ* Ap^r^	This study
PLB2299	CF7789 *csrA*::*kan* P2-*iraD*'*-*'*lacZ* Ap^r^ Km^r^	This study
PLB2484	CF7789 P2-*iraD*'*-*'*lacZ* (CsrA BS2, GGA to CGA) Ap^r^	This study
PLB2485	CF7789 P2-*iraD*'*-*'*lacZ* (CsrA BS3, GGA to GCA) Ap^r^	This study
PLB2492	CF7789 P1-P2-*iraD*'*-*'*lacZ* (CsrA BS2, GGA to CGA) Ap^r^	This study
PLB2493	CF7789 P1-P2-*iraD*'*-*'*lacZ* (CsrA BS1, GGA to GCA) Ap^r^	This study
PLB2495	CF7789 P2-*iraD*'*-*'*lacZ* (CsrA BS1, GGA to GCA) Ap^r^	This study
PLB2497	CF7789 P1-P2-*iraD*'*-*'*lacZ* (CsrA BS3, GGA to GCA) Ap^r^	This study
PLB2498	CF7789 P1-P2-*iraD*'*-*'*lacZ* (CsrA BS4, GGA to GCA) Ap^r^	This study
PLB2499	CF7789 P2-*iraD*'*-*'*lacZ* (CsrA BS4, GGA to GCA) Ap^r^	This study
PLB2605	CF7789 P*_lacUV5_-iraD*'*-*'*lacZ* Ap^r^	This study
PLB2606	CF7789 *csrA*::*kan* P*_lacUV5_-iraD*'*-*'*lacZ* Ap^r^ Km^r^	This study
PLB2609	CF7789 P1-*iraD-lacZ* Ap^r^	This study
PLB2610	CF7789 *csrA*::*kan* P1-*iraD-lacZ* Ap^r^ Km^r^	This study
PLB2611	CF7789 P2-*iraD-lacZ* Ap^r^	This study
PLB2612	CF7789 *csrA*::*kan* P2-*iraD-lacZ* Ap^r^ Km^r^	This study
PLB2638	CF7789 P1-ORF23′-'*lacZ* Ap^r^	This study
PLB2639	CF7789 P1-ORF47′-'*lacZ* Ap^r^	This study
PLB2649	CF7789 P1-*iraD*'-'*lacZ* (ORF47 start to stop, ATG to TAA) Ap^r^	This study
PLB2650	CF7789 P1-*iraD*'-'*lacZ* (ORF23 start to stop, ATG to TAA) Ap^r^	This study
PLB2651	CF7789 *csrA*::*kan* P1-P2-*iraD*'*-*'*lacZ* (CsrA BS2, GGA to CGA) Ap^r^ Km^r^	This study
PLB2668	CF7789 P1-P2-*iraD*'*-*'*lacZ* (ORF27 start to stop, ATG to TAG) Ap^r^	This study
PLB2671	CF7789 P1-P2-ORF27′-'*lacZ* Ap^r^	This study
PLB2672	CF7789 *csrA*::*kan* P1-P2-ORF27′-'*lacZ* Ap^r^ Km^r^	This study
PLB2674	CF7789 *csrA*::*kan* P1-P2-*iraD*'*-*'*lacZ* (ORF27 start to stop, ATG to TAG) Ap^r^ Km^r^	This study
S17-1 *λpir*	*recA thi pro hsdR-M + RP4*: *2-tc*::*mu km*::*tn7 λpir*^*+*^	31
TRCF7789	CF7789/*csrA*::*kan* Km^r^	7
TRMG1655	MG1655/*csrA*::*kan* Km^r^	7
FLAG-*iraD*	MG1655/*3xFLAG-iraD* Km^r^	This study
FLAG-*iraD csrA*	MG1655/*3xFLAG-iraD* Km^r^ *csrA*::*gm* Gm^r^	This study

a All fusions were integrated into the *λ att* site via the CRIM system (32). The *iraD* sequences in each fusion are indicated relative to the *iraD* translation initiation codon. P1-P2-*iraD*'*-*'*lacZ* translational fusions contain nt −498 to +20. P2-*iraD*'*-*'*lacZ* translation fusions contain nt −234 to +20. The P1-*iraD-lacZ* transcriptional fusion contains nt −547 to −413. The P2-*iraD-lacZ* transcriptional fusion contains nt −253 to −134. The P*_lacUV5_-iraD*'*-*'*lacZ* leader fusion contains nt −138 to +20. ORF23'*-*'*lacZ* translational fusions contain nt −498 to −376. ORF47'*-*'*lacZ* translational fusions contain nt −498 to −323. P1-P2-ORF27'-'*lacZ* translational fusions contain nt −498 to −58. Ap^r^, ampicillin resistance Gm^r^, gentamicin resistance; Km^r^, kanamycin resistance.

The C-terminal 3×FLAG-tagged *iraD* strain was constructed using the λ Red recombinase method as previously described (32). P1-mediated transduction was then used to introduce the *csrA*::*gm* allele into this strain. The *csrA*::*gm* allele was derived from the TRMG1655 *csrA*::*kan* strain, where the kanamycin marker was replaced with a gentamicin marker (12).

### β-Galactosidase assay.

Bacterial cultures containing *lacZ* fusions were grown at 37°C in Luria-Bertani (LB) broth supplemented with 100 μg/ml ampicillin and 50 μg/ml kanamycin for *csrA*::*kan* strains. Cells were harvested at various times throughout growth. β-Galactosidase activity was measured as described previously (19). At least three independent experiments were performed for each strain.

### Quantitative reverse transcriptase PCR (qRT-PCR).

qRT-PCR was carried out as previously described (33). Bacterial cultures grown to mid-exponential phase in LB at 37°C were added directly to RNAprotect Bacteria Reagent (Qiagen) according to the instructions of the manufacturer to stabilize RNA. Total RNA was purified with an RNeasy minikit (Qiagen), and DNA was removed with a Turbo DNA-free kit (Ambion). An IScript One-Step RT-PCR kit with SYBR green (Bio-Rad) was used to measure RNA or DNA control levels according to the manufacturer’s instructions. Reaction mixtures (20 µl) contained 100 ng of RNA (or an appropriate concentration of DNA standard), 1× SYBR green RT-PCR reaction mix, and a 300 nM concentration of each primer. The thermal cycling was done with an iCycler thermocycler (Bio-Rad) with 10 min reverse transcription at 50°C for 2 min at 95°C and then 45 cycles of PCR at 95°C for denaturation for 10 s and at 60°C for annealing, extension, and detection for 20 s. Melting curves were used to verify the specificity of the PCR product after the qRT-PCR reaction. ICycler iQ software (Bio-Rad) was used to determine RNA abundance relative to a standard curve of PCR products and 16S rRNA levels.

### Western blotting.

Western blotting followed previously published protocols (10). Bacterial cultures were grown in LB at 37°C, and samples were collected throughout growth. Cell pellets were resuspended in Laemmli buffer and lysed with boiling and sonication. Samples were separated by SDS-PAGE on 15% bis-Tris gels. Proteins were transferred to 0.2-µm-pore-size polyvinylidene difluoride membranes with electroblotting performed using a Mini Trans Blot system (Bio-Rad). 3×FLAG-tagged IraD was detected with anti-FLAG M2 monoclonal antibody (Sigma), and RpoB was detected with anti-RpoB monoclonal antibody (Neoclone). Horseradish peroxidase-linked ECL anti-mouse IgG antibody (GE Healthcare) and SuperSignal West Femto chemiluminescent substrate (Pierce) were used to detect signal. Images were collected using a ChemiDoc XRS+ system (Bio-Rad) and quantified using Quantity One software (Bio-Rad).

### Gel mobility shift assay.

Quantitative gel mobility shift assays followed a published procedure (14). His-tagged CsrA (CsrA-H6) was purified as described previously (34). WT and mutant RNAs started at the transcription initiation site from the *iraD* P2 promoter and were synthesized using an RNAMaxx kit (Agilent Technologies) and PCR-generated DNA templates. Gel-purified RNA was dephosphorylated and then 5′ end labeled using T4 polynucleotide kinase (New England Biolabs) and [γ-^32^P]ATP (7,000 Ci/mmol). Labeled RNAs were renatured by heating for 1 min at 85°C followed by slow cooling to room temperature. Binding reaction mixtures (10 µl) contained 0.1 nM labeled RNA, 10 mM Tris-HCl (pH 7.5), 10 mM MgCl_2_, 100 mM KCl, 32.5 ng of yeast RNA, 7.5% glycerol, 20 mM dithiothreitol, 4 U of RNase inhibitor (Promega), 0.1 mg/ml xylene cyanol, and various concentrations of purified CsrA-H6. Previously frozen CsrA was thawed and activated by incubation for 15 min at 37°C. Reaction mixtures were incubated for 30 min at 37°C to allow CsrA-RNA complex formation and then fractionated through 15% polyacrylamide gels. Free and bound RNA species were visualized with a Typhoon 9410 phosphorimager (GE Healthcare). CsrA-RNA interactions were quantified as described previously (14).

### Footprint assay.

CsrA-RNA footprint assays followed a published procedure (19). Labeled WT RNA beginning at the P2 transcription start site described for the gel mobility shift analysis was used. Additional WT and CsrA BS2 mutant RNAs started 17 nt upstream of the P2 transcription start site. Reaction mixtures were identical to those in the gel shift assay except that RNase inhibitor was omitted, the concentration of labeled RNA was increased to 10 nM, and 200 μg/ml acetylated bovine serum albumin (BSA) was added. After a 30-min incubation at 37°C to allow CsrA-RNA complex formation, RNase T1 (0.12 U) was added and incubation was continued for 15 min at 37°C. Reactions were stopped by adding 10 µl of Stop buffer (95% formamide, 0.025% SDS, 20 mM EDTA, 0.025% bromophenol blue, and 0.025% xylene cyanol) and cooling on ice. RNAs were fractionated through the use of standard 6% (vol/vol) polyacrylamide-8 M urea sequencing gels. Cleaved patterns were examined using a phosphorimager.

### Toeprint assay.

CsrA-RNA toeprint assays followed a published procedure (14). The *iraD* RNA sequence used for toeprinting extends from the *iraD* P2 transcription start site through +20 relative to the *iraD* translation initiation codon and includes a 3′ extension derived from plasmid vector sequence. Gel-purified *iraD* RNA (150 nM) was hybridized in Tris-EDTA (TE) buffer to a 5′-end-labeled DNA oligonucleotide (150 nM) complementary to the vector-derived 3′ extension by heating for 3 min at 85°C followed by slow cooling to room temperature. Toeprint reaction mixtures (10 µl) contained 2 µl of the hybridization mixture (30 nM final concentration), 1.5 µM CsrA-H6, a 375 µM concentration of each deoxynucleoside triphosphate (dNTP), 10 mM dithiothreitol (DTT), and avian myeloblastosis virus (AMV) reverse transcriptase buffer. Previously frozen CsrA was thawed and activated by incubation for 15 min at 37°C. Mixtures were incubated for 30 min at 37°C to allow CsrA-RNA complex formation. AMV reverse transcriptase (0.3 U) was then added, and incubation was continued for 15 min at 37°C. Reactions were terminated by the addition of 6 µl of gel loading buffer. Samples were fractionated through the use of standard 6% (vol/vol) polyacrylamide–8 M urea sequencing gels. Toeprint patterns were visualized with a phosphorimager.

### Coupled transcription-translation assay.

*In vitro* coupled transcription-translation assays using PURExpress (New England Biolabs) followed a published procedure (19). Plasmid pT7-P1*iraD*'*-*'*lacZ* contains a T7 promoter driving transcription of the translational fusion from the natural *iraD* P1 transcription start site (Fig. 1). Plasmid pT7-P1(BS2)*iraD*'*-*'*lacZ* is identical to pT7-P1*iraD*'*-*'*lacZ* except that it contains a GGA-to-CGA mutation in BS2. Similarly, plasmids pT7-P2*iraD*'*-*'*lacZ* and pT7-P2(BS2)*iraD*'*-*'*lacZ* contain a T7 promoter that drives transcription from the natural *iraD* P2 transcription start site. These four plasmids were used as templates for coupled transcription-translation reactions using the PURExpress *in vitro* protein synthesis kit according to the manufacturer’s instructions. Reaction mixtures contained a 20 nM concentration of plasmid DNA template and various concentrations of purified CsrA-His6 with 10 U of RNase inhibitor (Promega). The mixtures were incubated for 2 h at 37°C, and β-galactosidase activity was determined according to the manufacturer’s instructions.

### mRNA half-life analysis.

Cultures were grown at 37°C in LB broth to exponential phase prior to the addition of rifampin (200 µg/ml) to prevent transcription initiation. After 1 min following rifampin addition, 0.8-ml aliquots were removed at various times and total cellular RNA was prepared by the hot phenol extraction method. To measure the *iraD* mRNA half-life, total cellular RNA (30 µg) was annealed to 2 pmol of an *iraD*-specific 5′ end-labeled primer in a 25-µl reaction mixture. Reaction mixtures were renatured by heating for 1 min at 80°C and slowly cooling to 25°C. Primer extension procedures were performed according to the manufacturer’s instructions (Superscript III; Life Technologies, Inc.) for 2 h at 50°C. The resulting products were fractionized through the use of 8% (vol/vol) polyacrylamide-8 M urea sequencing gels. Quantitative analysis was performed using a phosphorimager.
