# Supplementary figures and images for: Translational Repression of the RpoS Antiadapter IraD by CsrA Is Mediated via Translational Coupling to a Short Upstream Open Reading Frame
Source: mBio. 2017 Aug 29;8(4):e01355-17. doi: 10.1128/mBio.01355-17 (PMC5574718; doi:10.1128/mBio.01355-17)

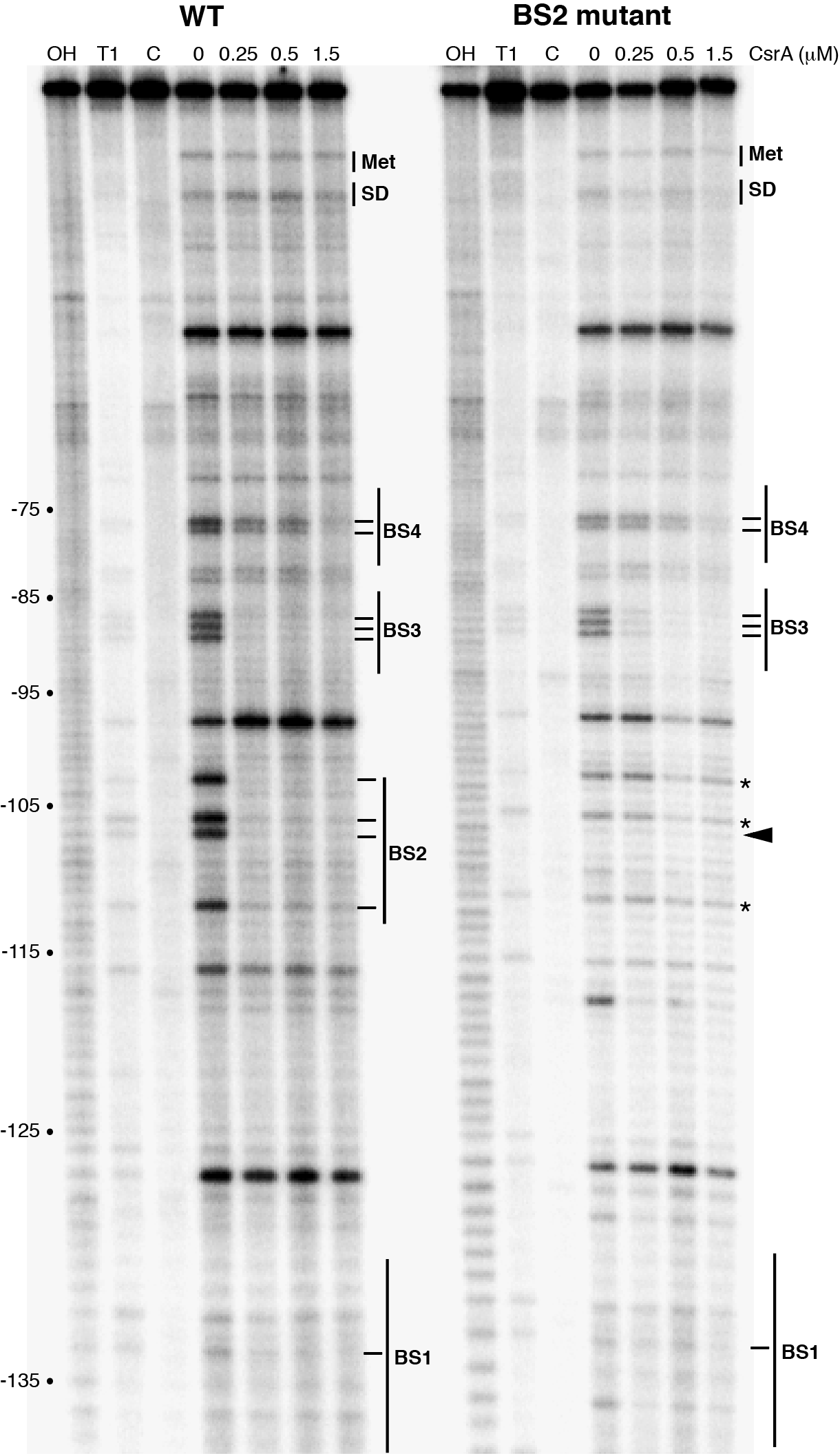

Supplement: FIG S1 [file mbo004173463sf1.tif]

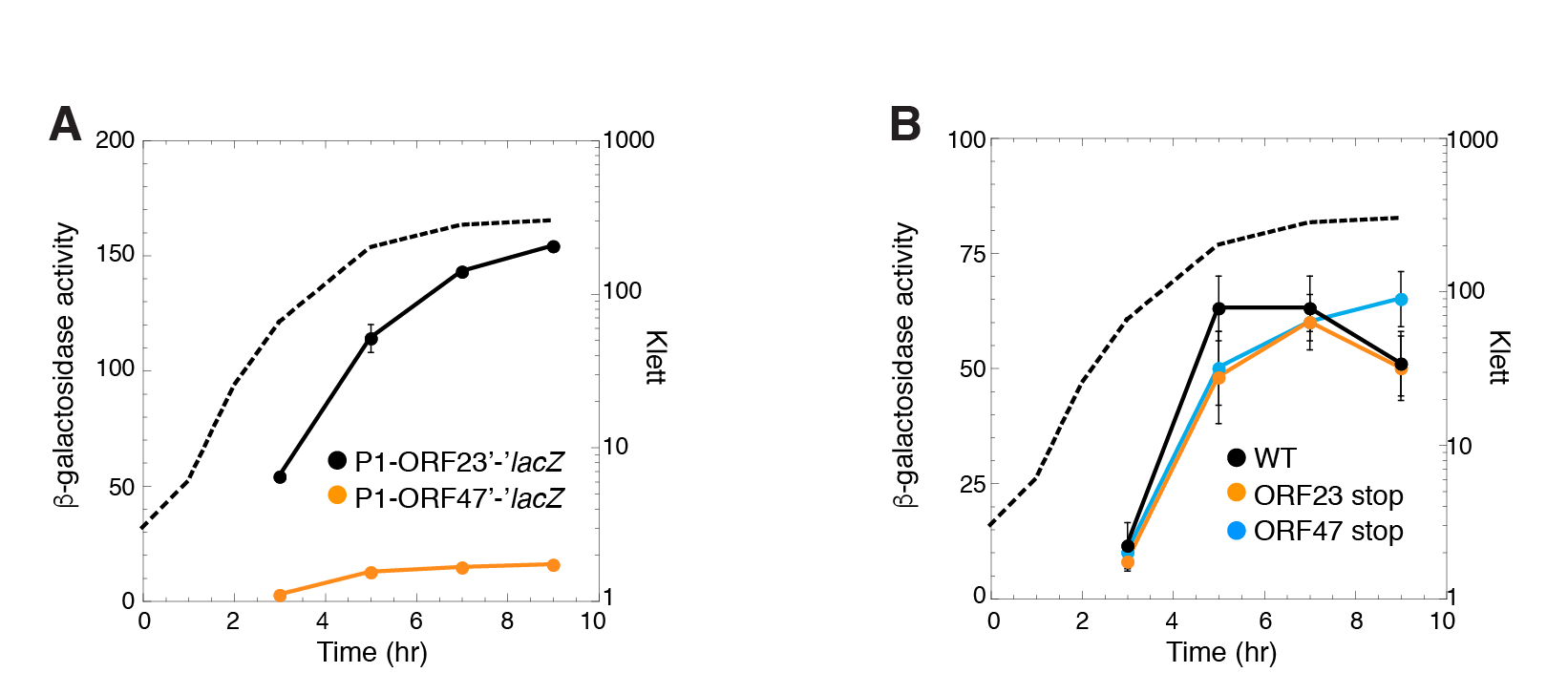

Supplement: FIG S2 [file mbo004173463sf2.tif]
